# A Review of Cutaneous Viral Infections and Their Potential Role in Neurologic Diseases

**DOI:** 10.3390/jcm14248770

**Published:** 2025-12-11

**Authors:** Valeria Duque-Clavijo, Hung Q. Doan, Stephen K. Tyring

**Affiliations:** 1 School of Medicine, Universidad de los Andes, Bogotá 111711, Colombia; v.duquec@uniandes.edu.co; 2 Department of Dermatology, Division of Internal Medicine, The University of Texas MD Anderson Cancer Center, Houston, TX 77030, USA; 3 Department of Dermatology, The University of Texas Health Science Center at Houston, Houston, TX 77030, USA; styring@ccstexas.com

**Keywords:** cutaneous viral infections, neuroinflammation, neurodegeneration, vaccination, herpesviruses

## Abstract

**Background:** Cutaneous viral infections, defined as viral pathogens that either primarily affect the skin (e.g., herpesviruses, enteroviruses) or frequently produce dermatologic manifestations despite systemic tropism (e.g., HIV, SARS-CoV-2), can trigger systemic inflammatory and neurotropic responses that extend their impact to the nervous system. A growing body of evidence suggests that viruses with dermatologic manifestations may play a significant role in the pathogenesis of neurologic disorders. **Summary:** Although individual viruses have been studied in isolation, the skin–brain axis in viral infections remains incompletely characterized. This review synthesizes existing knowledge and highlights gaps in understanding the mechanisms linking cutaneous viral infections to neurologic disease. We explore the principal mechanisms linking viral skin infections to central and peripheral nervous system damage, including direct neuroinvasion, immune-mediated injury, and vascular or endothelial dysfunction. Particular attention is given to herpesviruses, retroviruses, enteroviruses, and respiratory viruses, which have been associated with conditions such as dementia, multiple sclerosis, myelopathies, Guillain-Barré syndrome, and the post-acute neurologic sequelae of COVID-19. Furthermore, we discuss the role of neuroinflammation in viral-associated neurodegeneration and highlight emerging evidence supporting the recombinant zoster vaccine (Shingrix) as a potential modulator of neuroinflammatory processes and a protective factor against dementia. **Conclusions:** Cutaneous viral infections extend beyond local skin pathology, contributing to a broad spectrum of neurologic complications through intertwined infectious and inflammatory mechanisms. A clearer understanding of how peripheral viral activity shapes central nervous system vulnerability remains a major unmet need. A multidisciplinary approach integrating dermatologic and neurologic perspectives is essential for early recognition and prevention. While observational studies suggest that zoster vaccination may reduce viral reactivation and modulate neuroinflammatory pathways, definitive evidence of neuroprotection is still lacking. Future studies should clarify causal relationships, test mechanistic hypotheses regarding skin–brain immune crosstalk, and explore vaccine-mediated neuroprotection as a novel therapeutic strategy.

## 1. Introduction

Cutaneous viral infections represent a frequent challenge in dermatology, encompassing a broad clinical spectrum that ranges from self-limited exanthems to life-threatening systemic diseases. Beyond their cutaneous manifestations, increasing evidence indicates that several of these viruses possess neurotropic potential and can affect the central or peripheral nervous system through mechanisms such as direct neuronal invasion, immune-mediated injury, and virus-induced vascular inflammation.

Viruses commonly associated with skin lesions, including herpesviruses, enteroviruses, retroviruses, and respiratory viruses, may persist in latency or trigger inflammatory cascades extending beyond the skin. Although retroviruses and respiratory viruses are not traditionally considered dermatotropic pathogens, they were included in this review because they frequently produce clinically meaningful cutaneous manifestations and have well-established neurologic involvement. This dual dermatologic–neurologic relevance aligns with the scope of the present review, which focuses on viral pathogens at the intersection of skin disease and nervous system injury.

Their capacity to disrupt the blood–brain barrier, activate glial and endothelial cells, and induce systemic cytokine release underscores their contribution to neurologic sequelae ranging from acute encephalitis and Guillain–Barré syndrome to chronic neurodegenerative disorders such as dementia and multiple sclerosis. Despite strong evidence linking certain viruses to neurologic complications, an integrated dermatologic–neurologic framework remains lacking. The present review addresses this gap by examining how viruses with cutaneous manifestations contribute to CNS and PNS injury.

Understanding these skin–brain interactions is crucial for early recognition and preventive intervention. This review provides an integrative overview of the epidemiology, dermatologic features, and neuropathogenic mechanisms of cutaneous viral infections, emphasizing their clinical implications and the emerging neuroprotective role of vaccination.

## 2. Methods

This narrative review was conducted using PubMed, Embase, Scopus, and Google Scholar. Searches included the terms ‘cutaneous viral infections’, ‘neuroinvasion’, ‘herpesviruses’, ‘retroviruses’, ‘neurologic complications’, and ‘viral neuropathogenesis’. Articles published between 1990 and 2024 were included. Priority was given to systematic reviews, cohort studies, mechanistic studies, and high-impact original research. Non–peer-reviewed material was excluded. As a narrative review, no formal risk-of-bias tool was applied. Viruses were included if they demonstrated both cutaneous manifestations and documented neurologic involvement, even if they are not classically considered dermatotropic pathogens. This criterion explains the inclusion of retroviruses and respiratory viruses alongside traditional cutaneous viruses.

**Figure 1:**
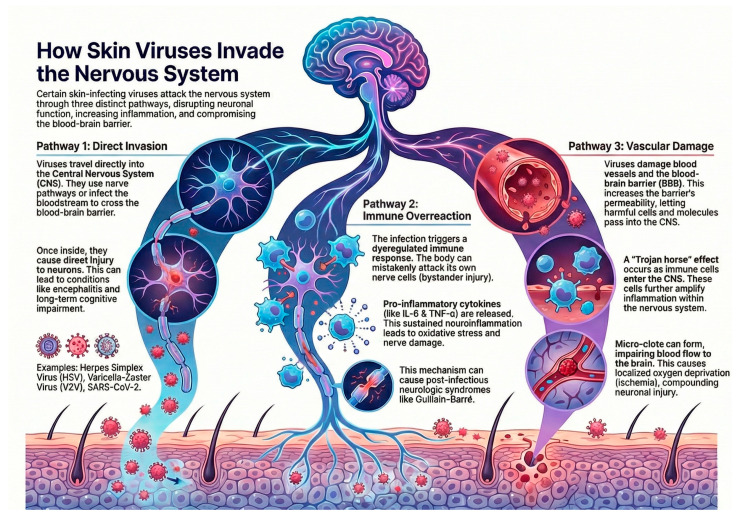
Mechanisms of neurologic involvement in cutaneous viral infections, illustrating direct neuroinvasion, immune-mediated injury, and vascular/endothelial pathways. Figure created by the authors using an AI-assisted illustration tool (NotebookLM; Google LLC, Mountain View, CA, USA; accessed on 22 November 2025).

Additionally, an AI-assisted illustration tool (Google NotebookLM; Google LLC, Mountain View, CA, USA; accessed on 22 November 2025) was used solely to generate the schematic layout for Figure 1; the authors provided all scientific content, verified accuracy, and performed all final editing of the figure.

## 3. Epidemiology and Dermatologic Spectrum of Cutaneous Viral Infections

A broad and expanding range of viruses are known to produce cutaneous manifestations, reflecting ongoing viral evolution, increased human mobility, and changing vector ecology. These factors contribute to the emergence of novel or previously uncommon viral exanthems worldwide [1,2].

Table 1 summarizes the principal viral families included in this review and their classic dermatologic manifestations. This table does not map individual skin phenotypes to neurologic outcomes; this omission is intentional and reflects a genuine gap in current clinical evidence, as phenotype–outcome associations remain poorly characterized. The neurologic implications of these pathogens are therefore addressed separately in subsequent sections.

## 4. Mechanisms of Neurologic Involvement in Cutaneous Viral Infections

Cutaneous viral infections can affect the nervous system through several interrelated mechanisms, each contributing to distinct patterns of neurologic injury. These pathways include:

(1) direct viral neuroinvasion,
(2) immune-mediated neuronal injury, and
(3) vascular and endothelial dysfunction.

Together, these mechanisms disrupt neuronal homeostasis, amplify neuroinflammation, and compromise the integrity of the blood–brain barrier. These pathways are best understood as conceptual models integrating current experimental and clinical evidence, as quantitative measurements (such as CSF viral titers or axonal transport kinetics) remain limited and pathogen-specific. These mechanisms are summarized schematically, as shown in Figure 1.

### 4.1. Direct Viral Neuroinvasion

Certain viruses reach the central nervous system (CNS) through retrograde axonal transport (e.g., HSV, VZV) or hematogenous spread, particularly when viremia leads to endothelial infection and BBB disruption, as seen with flaviviruses and SARS-CoV-2. Once inside the CNS, neurotropic viruses can cause neuronal injury via cell lysis, apoptosis, and synaptic dysfunction, ultimately contributing to encephalitis and long-term neurocognitive impairment [3].

### 4.2. Immune-Mediated Neuronal Damage

Viral infections may trigger dysregulated immune activation resulting in bystander neuronal injury. Mechanisms include molecular mimicry, autoantibody formation, and activation of microglia and astrocytes, which release proinflammatory cytokines such as IL-1β, IL-6, and TNF-α. This pathway underlies several post-infectious neurologic syndromes, including Guillain–Barré syndrome. Neurotropic RNA viruses are particularly potent activators of innate immune pathways, sustaining neuroinflammation and oxidative stress [4,5].

### 4.3. Vascular and Inflammatory Pathways

Systemic viral infections can induce endothelial dysfunction, microvascular injury, and pericyte impairment, all of which increase BBB permeability. SARS-CoV-2 exemplifies this mechanism: cytokine storms and endothelialitis facilitate immune-cell trafficking into the CNS, amplifying inflammation through a “Trojan horse” mechanism. Microthrombi formation and vascular inflammation compound neuronal injury by impairing cerebral perfusion and promoting local ischemia [6,7].

Given these distinct yet overlapping mechanisms, it is essential to examine the viral pathogens most commonly implicated in neurologic disease. Several viral families with cutaneous manifestations have been linked to both acute and chronic neurologic syndromes, including:

- Herpesviruses: Herpes simplex virus (HSV), varicella-zoster virus (VZV), and human herpesvirus 6 (HHV-6) have been associated with dementia, postherpetic neuralgia (PHN), viral pruritus, and prothrombotic complications [8,9,10,11]. In addition, Epstein–Barr virus (EBV) has been strongly linked to multiple sclerosis.
- Enteroviruses: Enterovirus 71 and poliovirus are associated with conditions such as acute flaccid paralysis and encephalitis [12].
- Retroviruses: Human immunodeficiency virus (HIV) and human T-lymphotropic virus type 1 (HTLV-1) contribute to HIV-associated neurocognitive disorders and HTLV-1–associated myelopathy/tropical spastic paraparesis [9,13].
- Respiratory viruses: Influenza virus, respiratory syncytial virus (RSV), and coronaviruses (including SARS-CoV-2) have been implicated in encephalitis, seizures, and Guillain–Barré syndrome, with long COVID now recognized as a cause of persistent neurologic sequelae [9,14].

Collectively, these viruses cause neurologic injury through a convergence of direct viral, immune-mediated, and vascular pathways. Understanding these mechanisms is critical for improving diagnostic precision, therapeutic interventions, and preventive strategies in both dermatology and neurology.

## 5. Viral Infections and Dementia: Pathophysiologic Insights and Emerging Evidence

Dementia encompasses a group of progressive neurodegenerative disorders characterized by cognitive decline, memory impairment, and loss of functional independence. Alzheimer’s disease (AD) represents the most prevalent form, followed by vascular dementia (VaD).

Neuroinflammation is a central pathogenic feature of dementia, driven by the interplay between central and peripheral immune responses. Within the brain, microglia and astrocytes play pivotal roles. Upon activation, these glial cells release proinflammatory cytokines such as IL-1, IL-6, and TNF-α, which promote neuronal injury and synaptic dysfunction. Sustained activation of these cells fosters amyloid-β deposition and tau hyperphosphorylation, ultimately leading to the hallmark neuropathologic features of AD [15,16,17,18].

Systemic inflammation further amplifies these processes. Age-related immune dysregulation of both innate and adaptive pathways contributes to chronic neuroinflammatory states. Peripheral immune cells, including T and B lymphocytes, may infiltrate the CNS and exacerbate neuroinflammation, thereby accelerating cognitive decline [16,19].

In recent years, infectious agents have emerged as potential contributors to dementia pathogenesis, with accumulating evidence implicating cutaneous viral infections as possible triggers. Among these, herpes zoster virus (HZV), HSV, and HHV-6 have been studied most extensively.

Epidemiologic studies on HZV have yielded mixed results, but several have demonstrated an increased risk of dementia following HZV infection. A nationwide cohort study from South Korea reported that HZV infection was associated with higher incidence of both AD and VaD, while a meta-analysis showed that herpes zoster ophthalmicus (HZO) conferred the greatest relative risk [20,21].

HSV, particularly HSV-1, has been more consistently associated with an increased risk of dementia. Studies have shown that HSV-1 seropositivity is linked to a higher risk of dementia, including AD. Additionally, antiviral treatment for HSV has been associated with a reduced risk of developing dementia.

HHV-6 has also been implicated in AD pathogenesis, with studies suggesting a role in neuroinflammation and disease progression [11].

Overall, these findings underscore the hypothesis that chronic viral latency and reactivation may act as modulators of neurodegenerative processes. Further mechanistic and longitudinal studies are warranted to clarify causality and therapeutic implications. However, these associations should be interpreted with caution. Most available data derive from observational designs, which cannot establish temporality or dose–response relationships. Alternative explanations, including reverse causation (early cognitive decline increasing susceptibility to infections), survival bias, and unmeasured confounders, may partly account for the observed links between herpesviruses and dementia.

## 6. Post-Herpetic Neuralgia and Virus-Associated Pruritus: Pathophysiology, Clinical Implications, and Management

Post-herpetic neuralgia (PHN) and virus-associated pruritus are significant complications following HZV infection, primarily caused by the varicella-zoster virus (VZV). The pathophysiology of PHN involves persistent neuroinflammation and nerve damage.

Mechanistic studies have revealed key molecular drivers of PHN. Experimental models of HSV-1 reactivation demonstrate that upregulation of protein arginine methyltransferase 6 (PRMT6) and inhibition of the cGAS–STING signaling pathway can sustain chronic neuroinflammation by attenuating antiviral innate immune responses [22]. Likewise, a CCL5/CCR5-mediated inflammatory cascade has been implicated in herpetic neuralgia, suggesting that CCR5 blockade may reduce pain via downregulation of proinflammatory cytokines [23].

Virus-associated pruritus, including postherpetic itch, is linked to neural sensitization and reduced intraepidermal nerve fibers, indicative of small fiber neuropathy [24]. Neuropathic itch mechanisms involve both peripheral and central nervous system dysfunctions, with treatments focusing on topical agents like capsaicin and systemic medications such as gabapentin [25,26].

Importantly, these mechanisms correlate with measurable clinical biomarkers. Reduced intraepidermal nerve fiber density on skin biopsy has been consistently documented in patients with postherpetic itch and PHN, supporting a structural small-fiber neuropathy endophenotype. Likewise, quantitative sensory testing (QST) frequently reveals thermal hypoesthesia and mechanical allodynia, which align with the molecular pathways of peripheral sensitization described above. However, not all molecular findings have validated clinical correlates, and this represents a key translational gap. Larger longitudinal studies integrating mechanistic biomarkers, QST profiles, and biopsy metrics are needed to clarify their diagnostic and prognostic utility.

Management of PHN includes antiviral therapy within 72 h of symptom onset to reduce acute pain and prevent PHN. Symptomatic treatments involve analgesics, tricyclic antidepressants, and antiepileptic drugs. For refractory cases, interventional treatments like epidural injections and pulsed radiofrequency of the dorsal root ganglion are considered [27].

## 7. Viral Infections and Multiple Sclerosis: Immunopathogenic Links and Emerging Evidence

The association between viral infections and the development of multiple sclerosis (MS) has been extensively studied, with Epstein–Barr virus (EBV) emerging as the most significant viral risk factor. Epidemiological evidence indicates that nearly all MS patients have a history of EBV infection, and the risk of developing MS increases markedly following infectious mononucleosis, a symptomatic primary EBV infection [28,29].

Mechanistically, EBV is thought to contribute to MS through several pathways, including molecular mimicry, where EBV antigens resemble myelin proteins, leading to an autoimmune response against the central nervous system [30]. Additionally, EBV can infect and persist in B cells, potentially reprogramming them and promoting chronic immune activation and autoreactivity [29].

Other viruses, such as HHV-6 and human endogenous retroviruses (HERVs), have also been implicated in MS pathogenesis. HHV-6 and HERVs may contribute to neuroinflammation and demyelination through similar mechanisms of molecular mimicry and chronic immune activation [31].

## 8. Viral Myelopathies: Mechanisms and Clinical Impact

Myelopathy refers to a disorder involving the spinal cord, characterized by motor, sensory, and autonomic dysfunction. It can be caused by various etiologies, including compressive, vascular, metabolic, toxic, infectious, autoimmune, neoplastic, and paraneoplastic factors [32,33].

HTLV-1 is a well-established etiological agent of myelopathy, specifically HTLV-1-associated myelopathy/tropical spastic paraparesis (HAM/TSP). This chronic progressive disorder is characterized by spastic paraparesis, urinary dysfunction, and mild sensory impairment [34,35].

Other viral infections have also been implicated in spinal cord pathology. HIV is associated with vacuolar myelopathy, a chronic, progressive spinal cord disease observed in advanced HIV infection, leading to gait disturbances, lower limb weakness, and sensory loss [36]. Additionally, Cytomegalovirus (CMV) can cause viral myelitis, particularly in immunocompromised individuals, often presenting with rapidly progressive flaccid paralysis and sensory deficits [37,38].

## 9. Guillain-Barré Syndrome and Viral Triggers

Guillain-Barré syndrome (GBS) is an acute, immune-mediated polyradiculoneuropathy characterized by rapidly progressive weakness and sensory deficits, often leading to paralysis. It is typically triggered by an antecedent infection, which induces an aberrant autoimmune response targeting peripheral nerves [39]. Among the various infectious agents linked to GBS, Epstein–Barr virus (EBV) and SARS-CoV-2 have been increasingly studied.

Regarding EBV, a study by Leonhard et al. found that serologic evidence of recent EBV infection was present in 1% of GBS patients in the International GBS Outcome Study cohort [40]. This suggests that while EBV is a recognized trigger, it is relatively rare compared to other infections. Similarly, Tam et al. confirmed the association between EBV infection and GBS in a nested case–control study, reinforcing the role of EBV as a potential antecedent infection [41]. The pathogenesis of EBV-associated GBS likely involves molecular mimicry, where the immune response to EBV antigens cross-reacts with peripheral nerve components, leading to demyelination or axonal damage. This mechanism is consistent with the broader understanding of GBS pathogenesis, as described by the American Academy of Neurology [42].

Although EBV is strongly associated with MS, these findings do not establish causation. Residual confounding, host genetic susceptibility, and methodological limitations in sero-epidemiologic research warrant cautious interpretation.

Beyond EBV, SARS-CoV-2 has also been implicated in GBS, particularly following the onset of the COVID-19 pandemic. Although the exact nature of this relationship remains under investigation. Several studies suggest an immune-mediated rather than direct viral mechanism, given the median onset of GBS symptoms 11–12 days after COVID-19. A systematic review by Uncini et al. (2020) reported 42 cases of GBS in the early pandemic, with most presenting as acute inflammatory demyelinating polyneuropathy (AIDP) and responding to intravenous immunoglobulin (IVIG) or plasma exchange [43]. A prospective cohort study by Luijten et al. (2021) found 22% of GBS patients had prior SARS-CoV-2 infection, showing a higher prevalence of the demyelinating subtype and facial palsy compared to historical controls [44]. Li et al. (2021) proposed a para-infectious mechanism, given the consistent timeframe of symptom onset [45].

## 10. Neurologic Sequelae of Long COVID

The neurologic sequelae of long COVID, also known as post-acute sequelae of SARS-CoV-2 infection (PASC), encompass a broad range of symptoms affecting both the central and peripheral nervous systems. Common neurologic manifestations include cognitive dysfunction (often referred to as “brain fog”), headache, dizziness, fatigue, anosmia (loss of smell), dysgeusia (loss of taste), myalgias, and sleep disturbances [46,47].

Cognitive impairments are particularly prevalent, with deficits in attention, working memory, processing speed, and verbal fluency being frequently reported. Structural brain changes, such as cortical thinning and white matter abnormalities, have been observed in patients with long COVID, correlating with these cognitive deficits [48]. Long-COVID neuroimaging studies vary widely in MRI sequences, acquisition parameters, and analytic pipelines, limiting comparability. Harmonized imaging standards are needed to improve reproducibility.

Additionally, there is an increased risk of mental health disorders such as depression, anxiety, and post-traumatic stress disorder (PTSD). Neurologic sequelae can also include episodic disorders like migraine and seizures, extrapyramidal and movement disorders, and peripheral neuropathy [46,48].

Cutaneous neurosensory symptoms such as paroxysmal diffuse burning and itching sensations have been reported in long COVID patients. These symptoms are associated with hypertrophy of nerve endings in the skin, suggesting a neuropathic basis for the dysesthesia experienced by these patients [46,48].

The pathophysiology of these neurologic symptoms is thought to involve mechanisms such as viral persistence, neuroinflammation, autoimmunity, coagulopathy, and endotheliopathy. These findings underscore the need for comprehensive and multidisciplinary management approaches to address the diverse and persistent neurologic symptoms experienced by long COVID patients [47,49].

## 11. Preventive Strategies and the Neuroprotective Role of Vaccination

The recombinant zoster vaccine (Shingrix), which contains the varicella zoster virus glycoprotein E, has been studied for its potential impact on dementia.

The Shingrix vaccine, also referred to as the recombinant zoster vaccine (RZV), is developed to prevent HZV (shingles) and its associated complications. This vaccine contains a single VZV glycoprotein, glycoprotein E (gE), combined with the AS01B adjuvant system. Glycoprotein E is the most abundantly expressed protein of VZV and plays a critical role in viral replication and cell-to-cell spread.

### 11.1. Mechanism of Action of the Recombinant Zoster Vaccine (Shingrix)

The immune response to the Shingrix vaccine begins with antigen presentation. Upon administration, the gE protein is recognized by the immune system as a foreign antigen [50,51]. The vaccine’s AS01B adjuvant system, composed of QS-21 (a saponin) and MPL (3-O-desacyl-4′-monophosphoryl lipid A), enhances the immune response by activating both innate and adaptive immunity. This adjuvant system facilitates the recruitment and activation of antigen-presenting cells (APCs), such as dendritic cells, which process and present the gE antigen to T cells [50,52].

Following antigen presentation, T cell activation occurs. The vaccine induces a robust CD4+ T cell response, characterized by the production of polyfunctional T cells that secrete cytokines, including IFN-γ, TNF-α, and IL-2. This cellular response plays a pivotal role in controlling VZV reactivation and establishing long-term immunity [53,54].

In addition to T cell activation, the vaccine stimulates B cells to produce high levels of anti-gE antibodies. These antibodies neutralize the virus and inhibit its spread within the host [51,55]. Furthermore, the vaccine promotes the recruitment of naive CD4+ T cells into the memory pool, thereby contributing to the durability of the immune response [56].

### 11.2. Potential Link Between Shingrix Vaccination and Reduced Dementia Risk

The inflammation seen in dementia, particularly AD, involves chronic neuroinflammation. This inflammatory milieu contributes to neuronal damage and the progression of neurodegenerative pathology. The Shingrix vaccine, comprising the varicella-zoster virus glycoprotein E (gE) and the AS01B adjuvant system, has demonstrated a potential to reduce the risk of dementia. This effect may be attributed to its ability to modulate the immune system, potentially mitigating the neuroinflammatory processes associated with dementia.

The mechanism by which Shingrix may exert this protective effect is multifaceted:

First, it reduces the likelihood of VZV reactivation. Shingrix prevents HZV (shingles) by boosting VZV-specific immunity, thereby limiting the reactivation of the virus. Evidence from an in vitro study indicates that VZV-infected cells in the outermost layer of brain blood vessels contain peptides associated with AD plaques, specifically amyloid-β42 and amylin [57]. By preventing VZV reactivation, the vaccine could potentially mitigate this pathological process. Additionally, VZV reactivation has been implicated in the reactivation of other latent viruses, such as HSV-1, which is strongly associated with AD pathology [58].

Second, the vaccine creates an immune modulation. The AS01B adjuvant in Shingrix enhances both innate and adaptive immune responses, leading to a robust and sustained activation of CD4+ T cells and the production of polyfunctional T cells. This immune modulation may help in maintaining a more balanced immune response, potentially reducing chronic neuroinflammation associated with dementia [50,59].

Third, the vaccine may provide a neuroprotective effect. Vaccination with Shingrix has been associated with a lower risk of dementia, possibly due to its ability to train the immune system to limit damaging inflammation. This nonspecific neuroprotection could be a result of the vaccine’s ability to enhance overall immune surveillance and reduce the inflammatory burden in the brain [60].

However, these mechanisms remain theoretical, and current evidence does not establish a causal pathway between Shingrix and dementia reduction. Observed associations may reflect unmeasured confounding or biases inherent to observational designs.

### 11.3. Clinical and Epidemiologic Evidence Supporting Neuroprotection

Recent studies have shown that Shingrix is associated with a decreased risk of dementia. A retrospective cohort study using data from the Optum Labs Data Warehouse found that full vaccination with Shingrix was associated with a significantly reduced risk of dementia compared to unvaccinated individuals [60].

Another study published in Nature Medicine confirmed these findings, showing that the recombinant shingles vaccine was associated with a lower risk of dementia compared to other vaccines commonly used in older adults, such as influenza and tetanus-diphtheria-pertussis vaccines. The study reported a 17% increase in diagnosis-free time for dementia in those vaccinated with Shingrix [60].

Additionally, a population-based cohort study within the UK Clinical Practice Research Datalink found that zoster vaccination was associated with a lower risk of dementia diagnosis [61].

A comparative analysis of the live attenuated shingles vaccine (ZVL) and the recombinant zoster vaccine (RZV) demonstrated that individuals vaccinated with RZV had a lower likelihood of developing dementia within the subsequent six years. This reduction in dementia risk corresponded to an additional 164 days without a dementia diagnosis [57].

However, the extent to which these associations reflect true neuroprotection remains uncertain. Most of the original studies do not report in detail how exposure time was handled or which covariates were included in adjusted models; therefore, key factors such as frailty, multimorbidity, socioeconomic status, and healthcare utilization remain unaccounted for. This lack of methodological transparency represents a major limitation and makes residual confounding highly likely.

Overall, current evidence supports an association rather than causation. Future randomized or quasi-experimental studies will be necessary to determine whether zoster vaccination confers a true neuroprotective effect.

## 12. Clinical Pathways: When Dermatologic Findings Should Prompt Neurologic Evaluation

Certain dermatologic presentations should prompt timely neurologic evaluation because they may signal central or peripheral nervous system involvement. These include ophthalmic-distribution zoster, recurrent HSV-1 flares accompanied by new cognitive, behavioral, or focal neurologic symptoms, VZV-associated radicular pain or progressive focal deficits, diffuse viral exanthems associated with acute confusion, severe headache, or meningismus, and post-viral dysesthesia or neuropathic pain that persists despite appropriate dermatologic management.

In these scenarios, early neurologic assessment is recommended, and when clinically indicated, neuroimaging (MRI preferred), cerebrospinal fluid (CSF) analysis, or electrodiagnostic testing should be performed to rule out CNS or PNS involvement. Clear referral pathways between dermatology and neurology may improve early detection of viral-associated neurologic complications.

## 13. Conclusions

Cutaneous viral infections extend far beyond localized skin involvement, contributing to a broad spectrum of neurologic complications. Through mechanisms such as direct neuroinvasion, immune-mediated damage, and disruption of the blood–brain barrier, these infections have been implicated in conditions including dementia, myelopathy, multiple sclerosis, Guillain-Barré syndrome, and the neurologic sequelae of long COVID. However, the precise sequence of events linking peripheral infection to central nervous system injury remains incompletely understood, highlighting an important knowledge gap.

Mounting evidence also supports a potential role for vaccines, notably the recombinant zoster vaccine, in reducing the risk of viral reactivation and mitigating the neuroinflammatory processes associated with dementia. Because current findings derive largely from observational studies, these associations should be interpreted with caution. Randomized trials and mechanistic studies are needed to determine whether vaccination confers true neuroprotective effects or reflects reduced viral burden and healthier baseline immune profiles.

Recognizing the interplay between viral infections, dermatologic manifestations, and neurologic disease emphasizes the need for interdisciplinary collaboration. Based on our synthesis, we propose the hypothesis that recurrent cutaneous viral reactivation functions as a peripheral amplifier of chronic neuroinflammation, particularly in individuals with underlying vascular or immunologic vulnerability. Future studies should continue to explore these connections to refine preventive, diagnostic, and therapeutic approaches and ultimately reduce the neurologic burden associated with viral infections.

## Figures and Tables

**Table 1:** Cutaneous Viral Pathogens: Classification, Genetic Material, Incubation Period, and Classic Dermatologic Manifestations.

Family	Genetic Material	Virus	Incubation Period (Days)	Classic Skin Findings
**Human Herpesvirus (HHV)**	DNA	Herpes Simplex Virus 1	3–14	Painful grouped vesicles on an erythematous base (often perioral)
		Herpes Simplex Virus 2	3–14	Painful grouped vesicles on genitals may ulcerate
		Varicella Zoster Virus (VZV)	3	Pruritic erythematous macules in crops following a dermatome, lesions are in all stages
		Epstein–Barr virus	30–50	Maculopapular rash. Small petechiae in hard or soft palate in 1/3 of patients
		Cytomegalovirus	21	Babies: purpuric macules and papules Immunocompromised: Lesions from vesicles to verrucous plaques
		HHV-6 (Roseola)	5–15	Macular to papular eruptions
**Enterovirus**	RNA—Positive sense	Coxsackievirus A	3–14	Macules, vesicles, yellow-to-gray ulcers with erythematous halo
		Coxsackievirus B		Maculopapular eruptions, may coalesce into a morbilliform pattern
		Poliovirus		Skin manifestations are uncommon
**Retroviruses**	RNA—Positive sense	Human Immunodeficiency virus—1 (HIV-1)	Years	Primary: papulosquamous exanthem Secondary: Infectious, neoplastic or noninfectious/nonneoplastic signs
		Human T-lymphotropic virus—1 (HTLV-1)		Infective dermatitis, association with T-cell lymphoma/leukemia
**Coronavirus**	RNA—Positive sense	SARS-CoV-2	5–12	Maculopapular lesions, Chilblain-like lesions

Adapted from Drago et al. (2021) [1] and Tyring (2002) [2].

## Data Availability

No new data were created or analyzed in this study. Data sharing is not applicable to this article.

## Abbreviations

The following abbreviations are used in this manuscript:

AD	Alzheimer’s disease
BBB	Blood–brain barrier
CNS	Central nervous system
EBV	Epstein–Barr virus
HZV	Herpes zoster virus
HSV	Herpes simplex virus
HHV-6	Human herpesvirus 6
MS	Multiple sclerosis
PHN	Postherpetic neuralgia
VZV	Varicella–zoster virus
